# The Principles and Practice of Modern Surgery

**Published:** 1844-09

**Authors:** 


*The Principles and Practice of Modern Surgery.* By ROBT. DRUITT, Surgeon. From the third London edition: illustrated with one hundred and fifty-three wood engravings, with notes and comments by JOSHUA B. FLINT, M. D., M. M. S. S., late Professor of Surgery, in the Medical Institute of Louisville. Philadelphia: LEA & BLANCHARD, 1844. pp. 568.

Again we are indebted to Lea & Blanchard, for an excellent English work, improved by the labors of an American Surgeon, and embellished with numerous additional engravings. We commend this treatise to our readers. To dentists, an elementary knowledge of the principles of surgery is indispensable. We extract from the "Provincial Medical Journal," the following notice of the work.

"It may be said with truth, that the 'Principles and Practice of Modern Surgery,' affords a complete though brief and condensed view of the entire field of modern surgery. We know of no work on the same subject, having the appearance of a manual, which includes so many topics of interest to the surgeon; and the terse manner in which each has been treated, evinces a most enviable quality of mind on the part of the author, who seems to have an innate power of searching out and grasping the leading facts and features of the most elaborate productions of the pen. Notwithstanding various weedings and alterations, we find that there are nearly fifty pages of additional matter in the present volume, and evidently much has been done by both author and publishers to sustain the reputation already acquired. The wood-cuts have been greatly increased in number, and the pencil and graver of William Bagg, have added brilliancy to this portion of the book.

"It is a useful hand-book for the practitioner, and we should deem a teacher of surgery unpardonable who did not recommend it to his pupils. In our own opinion it is admirably adapted to the wants of the student; and with congratulation to the author and publishers, for the latter deserve much credit for the handsome appearance of the volume, on the success of their undertaking. We have the present edition as a piquant portion of the ample store of knowledge which it is the good fortune of the rising youth in the profession to be so cheaply provided with in the present day."—*Prov. Medical Journal.*

